# Primary Subcutaneous Umbilical Endometriosis: Case Report and Review of the Literature

**DOI:** 10.1155/2020/8899618

**Published:** 2020-11-30

**Authors:** Lorenzo Capasso, Valerio Sciascia, Giuseppe Loiaco, Giovanni Guida, Francesco Iarrobino, Carmela Di Lillo, Salvatore Massa, Ferdinando Salzano de Luna

**Affiliations:** ^1^Unit of General and Oncologic Surgery, Department of Surgery, “Sant'Anna e San Sebastiano” Hospital of Caserta via Palasciano, 81100 Caserta, Italy; ^2^Unit of Day-Surgery, Department of Surgery, “Sant'Anna e San Sebastiano” Hospital of Caserta via Palasciano, 81100 Caserta, Italy

## Abstract

We report the case of a patient diagnosed with primary umbilical endometriosis intending to discuss the diagnostic and therapeutic management of this rare disease. A 45-year-old woman suffering from a painful swelling located in the umbilical region, with intact and normal cutaneous aspect, came to our attention. Ultrasonography of the umbilical region showed a nodule with a nonhomogeneous echotexture pattern. Partial omphalectomy was performed under local anesthesia in day care setting surgery. Histology confirmed the diagnosis of umbilical endometriosis. Pre- and postoperative clinical controls showed no evidence for other endometriosis localization. No medical treatment was administered. No signs of recurrence were observed after 5 years from surgery. A review of the literature of the last 10 years was generated based on MEDLINE research, selecting some specific keywords. Several lesions can occur in the umbilical region, and endometriosis has to be ruled out even in patients without any surgery in their medical history. Surgery is the gold standard treatment for this condition: partial and radical omphalectomy are the two treatment options. We believe that given the significant psychological and aesthetical value of the umbilicus, surgical treatment has to be tailored and in case of a small endometrial umbilical nodule, partial omphalectomy (local excision of the umbilical endometrial nodule) with a 3 mm free border, even without adjuvant hormonal treatment, could ensure adequate and effective treatment.

## 1. Introduction

Endometriosis is a benign condition characterized by endometrial tissue found in anatomical localizations other than the uterus. It has been estimated that the prevalence of endometriosis ranges up to 10% in the general female population: in particular, this condition mostly affects women in fertile age (up to 40% during reproductive years) [1]. Furthermore, more than 30% of chronic pelvic pain patients and 5% to 50% of infertile patients have endometriosis [2, 3]. Endometrial implants have been reported in many ectopic sites, abdominal wall included, where they normally occur in old surgical scars due to open uterine surgery, more often caesarean section. Among abdominal localizations, umbilical endometriosis (UE) is a very rare finding, accounting for about 0.5-1.0% of external endometriosis [4, 5]. Based on aetiology, UE can be classified as primary or secondary UE. Primary UE, also known as Villar's nodule (who first described this scenario in 1886), arises spontaneously with not completely unravelled pathogenetic mechanisms, and it represents almost 75% of all cases of UE [6]; secondary UE can result from laparoscopic or open surgical procedures with umbilicus involvement. Both primary and secondary UE can be found as cutaneous or subcutaneous lesions [7]. Here, we present the case of a patient diagnosed with primary subcutaneous umbilical endometriosis. The observation of this rare clinical case at our institution prompted us to review the literature to address possible therapeutic management.

## 2. Patient and Methods

A good-looking 45-year-old woman was admitted to our hospital, suffering from pain located at the umbilicus where a solid, well-circumscribed subcutaneous nodule was palpable on physical examination. The lesion size was approximately 1 × 1 cm, increasing during the menstrual cycle. The skin above the swelling appeared normal and intact. Medical history revealed a few years of cyclical pain during menstrual period, no surgical intervention nor caesarian section, and two vaginal deliveries.

To better investigate the umbilical swelling, we decided to perform ultrasonography (US) of the periumbilical region, which allowed the clinical diagnostic hypothesis of PUE. In particular, US showed, in the subcutaneous layer, a roundish well-circumscribed nodule with a nonhomogeneous echotexture in the umbilicus, located close (but not attached) to the underlying muscular fascia, with no connection to intraperitoneal structures or organs. Because of the characteristic clinical and US presentation, no further imaging was needed. Then, the patient underwent surgical excision of the subcutaneous tumefaction. The procedure was accomplished under local anesthesia; after infiltration of 5 mL of 1% mepivacaine, a periumbilical semilunar incision of about 2 cm is performed around the clinically appreciable swelling (lower right quadrant). With skin dethatched, a subcutaneous nodule of about 1 cm in diameter with a typical brownish red color was detected. This nodule appeared indissociable from the overlying skin but easily dissociable with the electrocautery from the underlying fascia. En bloc excision of the nodule along with the overlying skin was performed, with a free round margin of 0.3 cm. Closure of the muscular fascia was achieved with an absorbable running suture (2/0 Polyglactin). Skin closure was completed with a subcuticular running suture (3/0 Polyamide), as for reductive onphaloplasty, ensuring an excellent overall aesthetic results. The patient was discharged on the same day. Histology confirmed the diagnosis of PUE. The patient underwent a 6-month follow-up schedule. No medical treatment was administered before and after surgery. Five years after surgery, no signs of local recurrence were observed.

## 3. Discussion

PUE is an unusual manifestation of extragenital endometriosis and accounts for 30-40% of all cutaneous forms of endometriosis [8, 9]. Indeed, the peculiarity of the case herein described is that the patient was diagnosed with primary umbilical endometriosis, without any suspicion of concomitant pelvic endometriosis and in the absence of previous surgery for either gynaecological disorders or caesarian section. In the latter cases, umbilical endometriosis is defined as secondary UE, which is due to iatrogenic dissemination and subsequent implant of endometrial cells during either laparoscopic or open surgical procedures [6]. While the pathogenesis of secondary UE seems to be intuitive, it is harder to define the origin of PUE. In this regard, several mechanisms have been proposed: the retrograde menstruation theory, the embryonic remnant theory, the celomic metaplasia theory, the migration theory, or a combination of them [10]. The widely accepted Sampson's theory assumes that endometrial implants in the abdomen arise from retrograde menstruation of endometrial tissue through the fallopian tubes. The embryonic rest theory describes that cell rests arising from the Wolffian and Müllerian duct system can undergo transformation into differentiated endometrial tissue; the celomic metaplasia theory originates from the consideration that the peritoneum, the ovary, and the Müllerian system embryologically derive from the celomic mesothelium and, under some stimulus, hormonal, inflammatory, or traumatic agents, the cells of the peritoneum undergo metaplastic change to form endometrial tissue; the migration theory postulates the dissemination of endometrial cells from the uterus, through various routes (vascular and lymphatic) to new localizations, even in sites remote from the pelvis, where they may implant and proliferate. Moreover, familial predisposition and immune system disorders have been suggested to play a role in the pathogenesis [11].

The mean age at the time of PUE diagnosis is estimated around 35-38 years, reflecting the fact that it takes an extensive exposure to hormonal, metaplastic, and/or environmental factors before this condition reaches the clinical level. The mean elapse of time from the onset of symptoms to the final treatment has been estimated as 13.3 months [6]. In our case, both the mean age at onset and the elapse of time before surgery are relatively consistent with the data shown in the literature.

The clinical presentation of PUE can be variable, mainly depending on its localization in the cutaneous or subcutaneous plane. Classic clinical features include umbilical swelling with associated cyclical pain. Patients can even show a bluish-purple mass, accompanied by catamenial bleeding from the umbilicus with or without associated pain or tenderness concomitantly with the menstrual cycle. Besides, the condition can be completely asymptomatic in some cases, while in literature, cases with continuous chronic pain have been described [8].

In patients with UE, a clinical scenario and physical examination are mainstays for diagnosis. US, MRI, and CT scan can be helpful, in particular to investigate the anatomical relationships of the nodule with the surrounding tissues. A recent Japanese retrospective study has shown that for UE, none of the imaging technique was superior in sensitivity when compared to physical examination and that US, MRI, and CT were almost equivalent [12]. However, imaging could guide through differential diagnosis with umbilical lesions. Tables 1 and 2 display a representation of cutaneous and subcutaneous differential diagnosis of umbilical lesions [6, 13–15]. Given the high number of possible differential diagnoses, we believe that the professional profile of the general surgeon plays a crucial role in assisting patients with a suspected PUE diagnosis.

Histological confirmation is the current golden standard for PUE diagnosis, while initial assessment for clinical workup is primarily clinical. Fine-needle aspiration cytology (FNAC) can be supplementary, but inconclusive results have been reported as high as 75% [16]. Moreover, FNAC could expose patients to the risk of tissue material dissemination along the needle path. Furthermore, elevated levels of CEA and CA125 tumor markers may raise the suspicion of concomitant pelvic endometriosis lesions.

In our case, the clinical presentation, along with the US pattern revealing no invasion of the underlying structures, was considered sufficient to establish a presumptive diagnosis to initiate treatment.

The management of UE is not standardized. Generally, medical treatment using progesterone, danazol, norethisterone, and GnRH analogues is still under debate. Indeed, medical treatment can be effective in relieving symptoms temporarily, but recurrences are frequent after treatment cessation [17]. Thus, definitive treatment has to be surgery. The operative options are radical omphalectomy, with the potential removal of the underlying fascia and peritoneum, followed by plastic reconstruction and partial omphalectomy (local resection of the endometrial nodule), with umbilical sparing. Radical omphalectomy is the most frequently performed operation for umbilical endometriosis. Additionally, total removal of the umbilicus might be performed in cases of a large extension of the nodule, especially in patients with a long-lasting history of symptoms.

Local resection of the umbilical endometrial nodule should be achieved with adequate borders of healthy tissue. Respecting sufficient borders and the integrity of the nodule itself is vital to minimize local recurrences after surgery [18]. The literature reports 13-15% incidence of simultaneous pelvic endometriosis presence [19]. As such, although some authors suggest a concomitant laparoscopic pelvic evaluation, this approach is not mandatory and should be taken into account in cases with a high index of suspicion for pelvic endometriosis [6].

To provide the reader with a comprehensive and detailed picture of the state of the art and to compare therapeutic choices with prognosis, the MEDLINE database was searched using the keywords “primary umbilical endometriosis”, “primary endometriosis of umbilicus”, “spontaneous umbilical endometriosis”, and “Villar's nodule”. Open and English-written articles of the last 10 years discussing cases of PUE with a reported follow-up were selected. The results are shown in Table 3. Of the reported cases, only in 1 case, surgery was not the main treatment. Of all other cases, independently of the type of surgical and hormonal treatment, no recurrences were described: this is particularly relevant when it comes to aesthetic and psychological factors associated with total excision of the umbilicus, given its importance regarding sexuality. Moreover, hormonal treatment may interfere with the desire for pregnancy.

**Figure 1:**
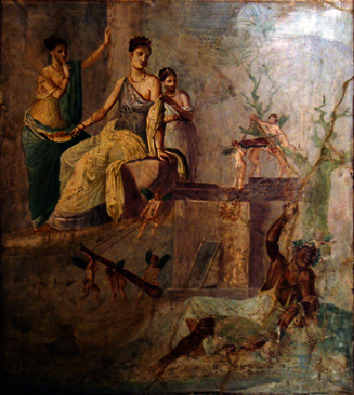
Omphale and Heracles (National Archaeological Museum, Naples).

The significance of the aesthetic and psychological value of the umbilicus requires tailored and therapeutic appropriateness. Since ancient Greece and in several old cultures, seductive power has been attributed to the umbilicus (from the Greek word “Omphale”). The Greek myth tells that Heracles, fascinated by the vision of the Queen of Lidia Omphale, bent to the will of the latter to the point that not only did he give her his club and lion skin but also he had himself reduced to a humble servant, carding her wool (Figure 1).

For each patient with a PUE diagnosis, the following factors should be considered:

1. Nodule size and relationship with the musculoaponeurotic fascia and the peritoneum
2. Other locations
3. Age of the patient
4. Desire for pregnancies
5. Aesthetic expectations

The clinical case we treated meets the efficacy and efficiency criteria because no recurrence was noticed during a 5-year follow-up period, and the patient kept a satisfactory aspect of the umbilicus. Before surgery, the patient only underwent an ultrasound examination which strengthened the diagnostic hypothesis of an endometrial nodule and specified its suprafascial position, allowing us to plan and carry out the intervention under local anesthesia and in the day care setting.

## 4. Conclusions

Based on our case and literature review, we believe that for small PUE nodules, without involvement of the musculoaponeurotic plane and other endometriosis localizations, partial omphalectomy (local excision of the umbilical endometrial nodule) with a 3 mm free border, even without adjuvant hormonal treatment, could ensure an adequate and effective treatment.

## Figures and Tables

**Table 1:** 

Differential diagnosis of umbilical cutaneous lesions
(i) Basal cell carcinoma
(ii) Dermatofibroma
(iii) Epidermoid cysts
(iv) Foreign body
(v) Hemangioma
(vi) Keloids
(vii) Melanoma
(viii) Neurofibroma
(ix) Primary umbilical endometriosis
(x) Pyogenic granuloma
(xi) Seborrheic keratosis
(xii) Umbilical polyp

**Table 2:** 

Differential diagnosis of umbilical subcutaneous lesions
(i) Abnormal embryonic development
(ii) Desmoid tumors
(iii) Endosalpingiosis
(iv) Lipoma
(v) Primary umbilical endometriosis
(vi) Sister Mary Joseph's nodule
(vii) Teratoma
(viii) Trichobezoar
(ix) Umbilical concretions
(x) Umbilical hernia
(xi) Urachal cysts and tumors

**Table 3:** Articles reporting cases of PUE. C: cutaneous; SC: subcutaneous; PO: partial omphalectomy; RO: radical omphalectomy; OC: oral contraceptives.

Authors	Year	No. of cases	Localization	Type of surgery	Medical treatment	Follow-up	Recurrence
Bagade and Guirguis [20]	2009	1	C	PO	No	6 weeks	No
Fernandes et al. [21]	2011	1	C	PO	No	12 months	No
Fancellu et al. [22]	2013	1	C	PO	OC	24 months	No
Ghosh and Das [14]	2014	1	C	PO	No	24 months	No
Pariza and Mavrodin [15]	2014	1	C	RO	Hormonal	6 months	No
Pramanik et al. [23]	2014	1	C	RO	No	6 months	No
Theunissen and IJpma [24]	2015	1	SC	PO	No	2 months	No
Taniguchi et al. [25]	2016	1	SC	RO	Dienogest, OC	12 months	No
Eğin et al. [26]	2016	1	C	RO	No	19 months	No
Brătilă et al. [27]	2016	1	SC	RO	No	3 months	No
Al-Quorain and Al-Yahya [28]	2017	1	C	RO	No	6 months	No
Loh et al. [29]	2017	1	C	PO	No	24 months	No
Chew et al. [30]	2017	1	C	No surgery	GnRH analogues and dienogest	13 months	No
Ouédraogo et al. [31]	2018	1	C	RO	No	12 months	No
Santos Filho et al. [32]	2018	6	C	RO	No	12-24 months	No
Capasso et al.	2020	1	SC	PO	No	5 years	No
